# Deoxynivalenol Has the Capacity to Increase Transcription Factor Expression and Cytokine Production in Porcine T Cells

**DOI:** 10.3389/fimmu.2020.02009

**Published:** 2020-08-13

**Authors:** Eleni Vatzia, Alix Pierron, Anna Maria Hoog, Armin Saalmüller, Elisabeth Mayer, Wilhelm Gerner

**Affiliations:** ^1^Institute of Immunology, Department of Pathobiology, University of Veterinary Medicine Vienna, Vienna, Austria; ^2^BIOMIN Research Center, Tulln, Austria

**Keywords:** deoxynivalenol, pig, CD4^+^ T cells, CD8^+^ T cells, γδ T cells, T-bet, Interferon-γ

## Abstract

Deoxynivalenol (DON) is a *Fusarium* mycotoxin that frequently contaminates the feed of farm animals. Pigs with their monogastric digestive system are in particular sensitive to DON-contaminated feed. At high concentrations, DON causes acute toxic effects, whereas lower concentrations lead to more subtle changes in the metabolism. This applies in particular to the immune system, for which immunosuppressive but also immunostimulatory phenomena have been described. Research in human and rodent cell lines indicates that this may be partially explained by a binding of DON to the ribosome and subsequent influences on cell signaling molecules like mitogen-activated protein kinases. However, a detailed understanding of the influence of DON on functional traits of porcine immune cells is still lacking. In this study, we investigated the influence of DON on transcription factor expression and cytokine production within CD4^+^, CD8^+^, and γδ T cells *in vitro*. At a DON concentration, that already negatively affects proliferation after Concanavalin A stimulation (0.8 μM) an increase of T-bet expression in CD4^+^ and CD8^+^ T cells was observed. This increase in T-bet expression coincided with elevated levels of IFN-γ and TNF-α producing T-cell populations. Increases in T-bet expression and cytokine production were found in proliferating and non-proliferating T cells, although increases were more prominent in proliferating cell subsets. Differently, IL-17A production by CD4^+^ T cells was not influenced by DON. In addition, frequencies of regulatory T cells and their expression of Foxp3 were not affected. In γδ T cells, GATA-3 expression was slightly reduced by DON, whereas T-bet levels were only slightly modulated and hence IFN-γ, TNF-α, or IL-17A production were not affected. Our results show for the single-cell level that DON has the capacity to modulate the expression of transcription factors and related cytokines. In particular, they suggest that for CD4^+^ and CD8^+^ T cells, DON can drive T-cell differentiation into a pro-inflammatory type-1 direction, probably depending on the already prevailing cytokine milieu. This could have beneficial or detrimental effects in ongoing immune responses to infection or vaccination.

## Introduction

Deoxynivalenol (DON) is a type B trichothecene mycotoxin produced by *Fusarium* fungi and contaminates cereal-based foods worldwide (1). As a result, it affects farm animals and in particular pigs, which are highly exposed to mycotoxins because of their cereal-rich diet. This frequently leads to health problems in this species (2, 3). *In vitro* studies performed on porcine lymphocytes and other immune-related cells have shown that DON impairs the function of these cells, such as their survival, proliferation and maturation (4–7). In a recent study, we could show by flow cytometry (FCM) phenotyping that DON concentrations higher than 0.4 μM decrease the proliferation of major porcine T-cell subsets, namely CD4^+^, CD8^+^, and γδ T cells (5). The same work revealed that DON concentrations above 0.4 μM have a negative impact on the expression of the co-stimulatory molecules CD27 and CD28, which are essential for optimal T-cell activation, proliferation and survival (8, 9).

In crystallography studies it was found that DON binds on the A-site of the 60S unit of the ribosome (10). Based on parallel findings it is assumed that this is involved in the activation of various mitogen-activated protein kinases (MAPKs), which results in immunostimulatory or immunosuppressive effects depending on the frequency, dose and the duration of exposure to the mycotoxin (11–14). Having identified the negative impact of DON on co-stimulatory molecules for T cells (see above), we hypothesized that DON might also influence the expression of transcription factors which are frequent targets of MAPK signaling (15).

Hence, in this study we investigated the influence of DON and its less toxic microbial transformation product deepoxy-deoxynivalenol (DOM-1) (16, 17) on the expression of three transcription factors involved in T-cell differentiation: T-bet, GATA-3, and Foxp3. We also analyzed the production of major cytokines produced by differentiated T cells, namely IFN-γ, TNF-α, and IL-17A. T-bet is a transcription factor that belongs to the T-box family and is described as the master regulator of Th1 differentiation (18, 19). Its expression is required for IFN-γ production in CD4^+^ and CD8^+^ T cells (20). For CD8^+^ T cells, it also promotes the function and longevity of memory cells (18). The transcription factor GATA-3 is involved in T-cell development and functional differentiation. Studies have shown that GATA-3 is essential for all stages of T-cell development in the thymus (21). GATA-3 has been also described as a master regulator of Th2 cell differentiation of CD4^+^ T cells and is necessary for Th2 cytokine gene expression with Th2 cells producing mainly IL-4, IL-5, and IL-13 (22).

Naïve CD4^+^ T cells can also differentiate into regulatory T cells (Tregs) and Th17 cells (23). Tregs have a crucial role in maintaining immune tolerance (24). The transcription factor Foxp3 is required for the thymic development and function of peripheral Tregs and thus is described as the master regulator of this cell type. Th17 cells that produce IL-17 are dependent on the transcription factor ROR-γt and in mice and humans it has been shown that they are involved in the clearance of extracellular pathogens (23). In pigs, the functional properties of these cells and their responses have been studied in bacterial *in vivo* studies (25), but currently no antibodies are available to study ROR-γt expression on the protein level (26).

Our results indicate that in the presence of T-cell receptor (TCR) stimulation via Concanavalin A (ConA), DON concentrations of 0.8 μM result in an upregulation of T-bet and to a lesser extent GATA-3, but not Foxp3. Increased T-bet expression levels coincided with increased frequencies of IFN-γ and TNF-α producing CD4^+^ and CD8^+^ T cells. Hence, we elucidate functional pathways for some of the described immuno-stimulatory capacities of DON.

## Materials and Methods

### Animals and Cell Isolation

Blood was collected into cups prefilled with a heparin solution (400 U/mL, Serva, Heidelberg, Germany, in PBS, PAN Biotech, Aidenbach, Germany). Six-month old healthy pigs from an abattoir served as blood donors. The animals were anesthetized with high electric voltage, which was followed by exsanguination, a procedure, which is in accordance to the Austrian Animal Welfare Slaughter Regulation.

Peripheral blood mononuclear cells (PBMCs) were isolated after density gradient centrifugation for 30 min at 920 × *g* (Pancoll human, density: 1.077 g/mL, PAN Biotech) as described before (27). Cells were counted by using a Cell Counter (XP-300 Hematology Analyzer, Sysmex Europe GmbH) before cryopreserving them at −150°C for future use. Freezing and thawing of PBMCs was performed as described elsewhere (28).

### *In vitro* Cultivation and Stimulation

Thawed PBMCs were counted in PBS and then labeled with the CellTrace^TM^ Violet Cell Proliferation Kit (Thermo Fisher Scientific, Waltham, MA, United States) as described elsewhere (29). Thereafter, cells were counted and 5 × 10^5^ cells per well were plated in 96-well round-bottom plates (Greiner Bio-One, Frickenhausen, Germany). PBMCs were cultivated in culture medium consisting of RPMI-1640 (PAN Biotech), including 10% fetal calf serum FCS (Sigma-Aldrich, Schnelldorf, Germany), 100 IU/ml penicillin (PAN-Biotech) and 0.1 mg/ml P/S streptomycin (PAN-Biotech). For the induction of a polyclonal T-cell stimulation, PBMC cultures were supplemented with ConA (3 μg/mL, Amersham Biosciences, Uppsala, Sweden) at 37°C and 5% CO_2_ for 4 days. To test the influence of DON and its metabolite deepoxy-deoxynivalenol (DOM-1), some ConA-stimulated cultures received DON at concentrations of 0.2 and 0.8 μM or DOM-1 at 16 μM. Experiments were performed with PBMCs from one individual pig for each experimental setup (see below). In total, PBMCs from six different pigs were tested in separate experiments. DON and DOM-1 were dissolved in sterile water to obtain a stock solution of 5 mM and stored at −20°C. Both substances were provided from Biopure, Romer Labs^®^, Tulln, Austria and had a purity of ≥99%.

### Flow Cytometry Staining for Transcription Factor Expression

After 4 days of *in vitro* cultivation, cells were harvested and washed in PBS + 3% FCS. The antibodies (Abs) listed in Table 1 were used for cell surface and intracellular staining in FCM. The staining was performed in 96-well round bottom plates; for the cell surface staining three consecutive incubation steps were carried out: primary Abs, secondary Abs and a third incubation with mouse IgG molecules (2 μg per sample, ChromPure, Jackson ImmunoResearch, West Grove, PA, United States; blocking of the free binding sites of the secondary Abs) and the viability dye VDeFluor780 (Thermo Fisher Scientific). Each incubation lasted for 20 min at 4°C and was always completed by two washing steps with PBS + 3% FCS. Thereafter, the cells were fixed and permeabilized with eBioscience^TM^ Foxp3/Transcription factor staining buffer set (Thermo Fisher Scientific) and were then incubated for 30 min with the directly conjugated mAbs for the transcription factors (Table 1). After the last incubation, the cells were washed in Perm/Wash buffer included in the buffer set and analyzed in 200 μL of the same buffer by a FACSCanto II flow cytometer (BD Biosciences, San Jose, CA, United States). At least 2 × 10^5^ lymphocytes were recorded per sample. The obtained FCM data were further analyzed by FlowJo software version 10.5.3 (FlowJo LLC, Ashland, OR, United States).

**TABLE 1:** Antibodies used for analysis of transcription factor expression in T-cell subsets.

Antigen	Clone	Isotype	Fluorochrome	Labeling strategy	Source of primary Ab
**Transcription factor expression in CD4^+^ T cells**					
CD4	74-12-4	IgG2b	Alexa488	Secondary antibody^a^	In house
CD8α	11/295/33	IgG2a	PE-Cy7	Secondary antibody^b^	In house
T-bet	eBio4B10	IgG1	PE	Directly conjugated	eBioscience
GATA-3	TWAJ	IgG2b	PerCP-eFluor710	Directly conjugated	eBioscience
**Transcription factor expression in Tregs**					
CD4	74-12-4	IgG2b	Alexa488	Secondary antibody^a^	In house
CD25	3B2	IgG1	Alexa647	Secondary antibody^c^	In house
Foxp3	FJK-16s	IgG2a	PE	Directly conjugated	eBioscience
CD8α	11/295/33	IgG2a	PE-Cy7	Secondary antibody^b^	In house
**Transcription factor expression in CD8β^+^ T cells**					
CD8β	PG164a	IgG2a	PE-Cy7	Secondary antibody^b^	Kingfisher Biotech
T-bet	eBio4B10	IgG1	PE	Directly conjugated	eBioscience
**Transcription factor expression in γδ T cells**					
TCR-γδ	PPT16	IgG2b	Alexa488	Secondary antibody^a^	In house
CD2	MSA4	IgG2a	PE-Cy7	Secondary antibody^b^	In house
T-bet	eBio4B10	IgG1	PE	Directly conjugated	eBioscience
GATA-3	TWAJ	IgG2b	PerCP-eFluor710	Directly conjugated	eBioscience

*^*a*^Goat anti-mouse IgG2b-Alexa488, Jackson Immuno Research. ^*b*^Goat anti-mouse IgG2a-PE-Cy7, Southern Biotech. ^*c*^Goat anti-mouse IgG1-Alexa647, Thermo Fisher Scientific.*

### Intracellular Cytokine Staining (ICS)

After 4 days of *in vitro* cultivation under the conditions mentioned above but before harvesting, cells were stimulated for 4 h with phorbol 12-myristate 13-acetate (PMA, 50 ng/mL, Sigma-Aldrich, Schnelldorf, Germany) and ionomycin (500 ng/mL, Sigma-Aldrich) combined with Brefeldin A (1 μg/mL, BD GolgiPlug^TM^, BD Biosciences). This combined stimulation by ConA (for 4 days) and PMA/ionomycin (for the final 4 h) was applied in order to induce T-cell activation and proliferation at the beginning of the cultivation by ConA, whereas PMA/ionomycin stimulation was applied to induce cytokine production. Although the combination of PMA and ionomycin is very potent for T-cell activation and proliferation, it also induces T-bet expression in human (30, 31) and porcine T cells (our unpublished findings). Hence, a sole activation by PMA/ionomycin would have interfered with the analysis of T-bet expression. Following this combined stimulation, the cells were harvested and subjected to FCM staining for the detection of intracellular cytokines. Abs used are listed in Table 2. For cell surface staining, three consecutive incubation steps were performed with the same conditions as for the transcription factor staining (see above). After the surface staining, the cells were fixed and permeabilized with BD Cytofix/Cytoperm and after 20 min were washed twice with BD Perm/Wash (both by BD Biosciences, San Jose, CA, United States). Finally, cells were incubated for 30 min with directly labeled monoclonal Abs against cytokines (see Table 2). After the last incubation, the cells were washed in BD Perm/Wash included in the kit and were analyzed in 200 μL of the same buffer by a FACSAria (BD Biosciences). At least 5 × 10^5^ lymphocytes were recorded per sample. The obtained FCM data were further analyzed by FlowJo software version 10.5.3 (FlowJo LLC, Ashland, OR, United States).

**TABLE 2:** Antibodies used for intracellular cytokine staining in T-cell subsets.

Antigen	Clone	Isotype	Fluorochrome	Labeling strategy	Source of primary Ab
**ICS in CD4^+^ T cells**					
CD4	74-12-4	IgG2b	Alexa488	Secondary antibody^b^	In house
CD8α	11/295/33	IgG2a	PE-Cy7	Secondary antibody^c^	In house
IFN-γ^a^	CC302	IgG1	Alexa647	Directly conjugated	Bio-Rad
TNF-α	Mab11	IgG1	BV605	Directly conjugated	BioLegend
IL-17A^a^	SCPL1362	IgG1	Alexa647	Directly conjugated	BD Biosciences
**ICS in CD8β^+^ T cells**					
CD8β	PG164a	IgG2a	PE	Secondary antibody^d^	Kingfisher Biotech
IFN-γ	CC302	IgG1	Alexa647	Directly conjugated	BioRad
TNF-α	Mab11	IgG1	BV605	Directly conjugated	BioLegend
**ICS in γδ T cells**					
TCR-γδ	PPT16	IgG2b	Alexa488	Secondary antibody^b^	In house
CD2	MSA4	IgG2a	PE-Cy7	Secondary antibody^c^	In house
TNF-α	Mab11	IgG1	BV605	Directly conjugated	BioLegend
IL-17A	SCPL1362	IgG1	Alexa647	Directly conjugated	BD Biosciences
IFN-γ	P2G10	IgG1	PE	Directly conjugated	BD Biosciences

*^*a*^Stained in parallel samples. ^*b*^Goat anti-mouse IgG2b-Alexa488, Jackson Immuno Research. ^*c*^Goat anti-mouse IgG2a-PE-Cy7, Southern Biotech. ^*d*^Goat anti-mouse IgG2a-PE, Southern Biotech.*

### Statistical Analysis

Descriptive statistics were performed by using GraphPad Prism V8.3 (GraphPad Software, San Diego, CA, United States). The data sets were subjected to multiple comparison tests with one-way ANOVA and Bonferroni’s multiple comparisons test. *p*-values ≤ 0.05 were considered as significant.

## Results

### Influence of DON on Transcription Factor Expression and Cytokine Production in Porcine CD4^+^ T Cells

**FIGURE 1:**
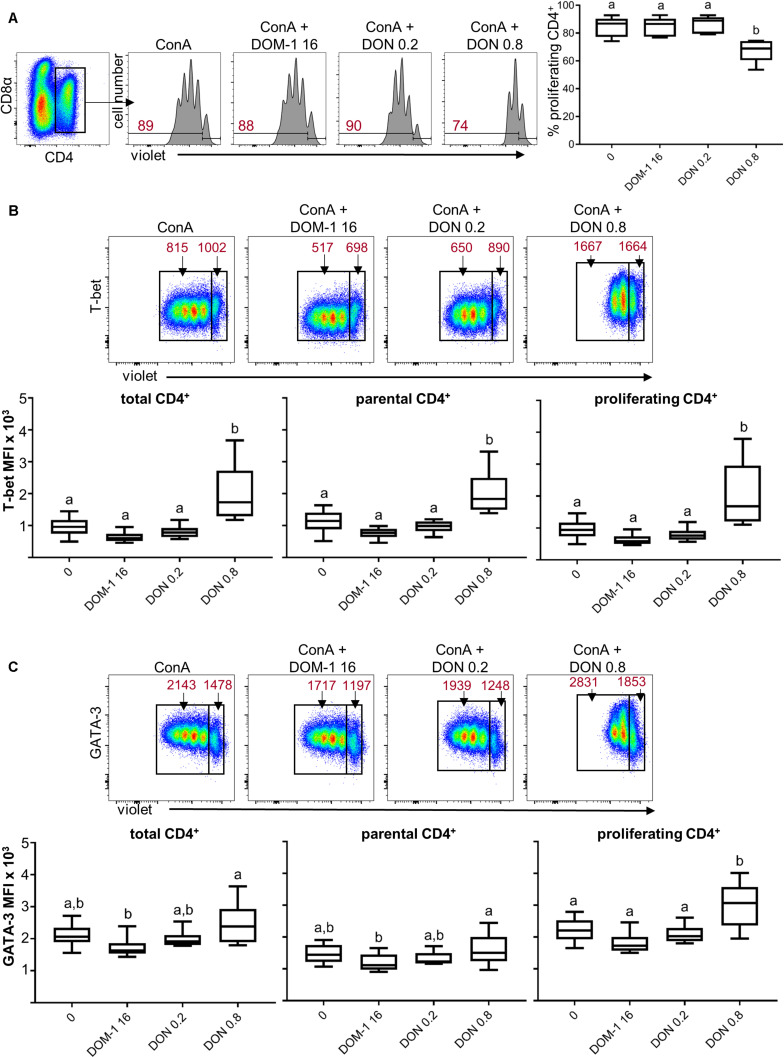
Proliferation and expression of T-bet and GATA-3 in CD4^+^ T cells in the presence of DON and DOM-1. Violet proliferation dye-stained PBMC were cultivated for 4 days in the presence of ConA alone or in combination with two different DON concentrations (0.2 and 0.8 μM) and DOM-1 (16 μM). After harvest, they were labeled for CD4, CD8α, T-bet, and GATA-3. **(A)** Flow cytometry panel: representative gating of total CD4^+^ T cells and raw data of proliferating CD4^+^ T cells under the different conditions. Red numbers show the percentage of proliferating cells. The boxplot on the right displays the percentage of proliferating CD4^+^ T cells for the different DON and DOM-1 conditions with PBMCs of six different pigs. **(B)** Analysis of T-bet expression: flow cytometry pseudocolor plots show representative raw data and gates that were applied to identify proliferating (cells gated on the left) and non-proliferating (parental, cells gated on the right) cells within CD4^+^ T cells. Red numbers indicate median fluorescence intensity (MFI) values for proliferating and non-proliferating cells. Box plots show T-bet expression levels as MFI for total (left), parental (middle), and proliferating (right) CD4^+^ T cells present in PBMCs of six different pigs. **(C)** Analysis of GATA-3 expression as in **(B)**. Different letters on boxplots indicate significant differences (*p* < 0.05).

We could previously show that DON at a concentration of 0.8 μM reduces proliferation of ConA-stimulated porcine T cells but does not completely abolish cell division (5). Hence, we assumed that this could be a concentration where DON might be able to induce immunomodulatory effects. PBMC were stimulated with ConA for 4 days in the absence or presence of DON at two different concentrations (0.2 and 0.8 μM) and DOM-1 (16 μM). After 4 days, cells were harvested and subjected to FCM analysis. In all experiments, blast cells were gated based on light scatter properties and dead cells were excluded from analysis as shown in Supplementary Figure 1A. Total CD4^+^ T cells were gated and their proliferation was analyzed (Figure 1A). As reported recently (5), a DON concentration of 0.8 μM led to a significant reduction in proliferation compared to the other conditions tested. In parallel to proliferation, expression of T-bet was analyzed in all CD4^+^ T cells (designated as “total”) as well as in non-proliferating (“parental”) and proliferating cells (Figure 1B). At 0.8 μM DON, expression of T-bet was significantly enhanced in total, parental and proliferating CD4^+^ T cells in comparison to the other three conditions (control without DON or DOM-1, 16 μM DOM-1, 0.2 μM DON). In parallel samples, GATA-3 expression was analyzed (Figure 1C). In proliferating CD4^+^ T cells, GATA-3 expression was found to be significantly elevated during the presence of DON at a concentration of 0.8 μM in comparison to the other three conditions. For total and parental CD4^+^ T cells, a significant increase was found for 0.8 μM of DON versus 16 μM of DOM-1.

**FIGURE 2:**
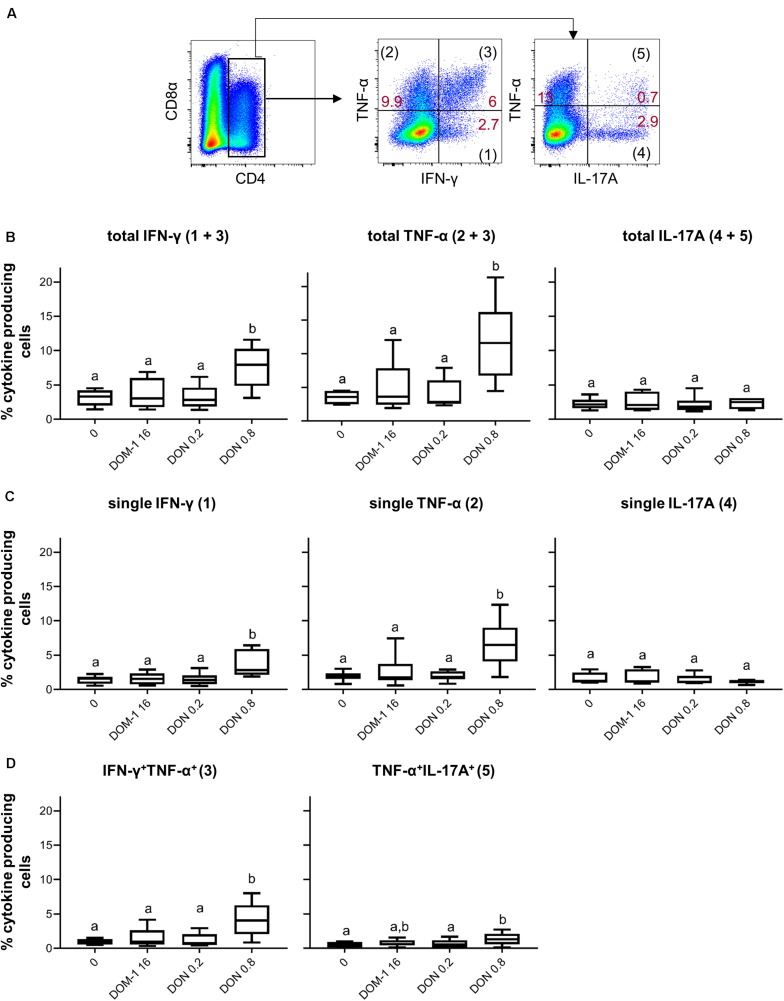
Frequencies of IFN-γ, TNF-α, and IL-17A producing CD4^+^ T cells in the presence of DON and DOM-1. Violet proliferation dye-stained PBMCs were cultivated for 4 days in the presence of ConA alone or in combination with two different DON concentrations (0.2 and 0.8 μM) and DOM-1 (16 μM). Following PMA/Ionomycin stimulation for 4 h on the fourth day, the cells were harvested and analyzed for cytokine production. **(A)** Total CD4^+^ T cells were gated and analyzed for production of IFN-γ, TNF-α, and IL-17A. IFN-γ single producing T cells are designated with (1), single TNF-α with (2), total IFN-γ with (1 + 3), total TNF-α with (2 + 3), double IFN-γ^+^TNF-α^+^ with (3), single IL-17A with (4), total IL-17A with (4 + 5), and double TNF-α^+^IL-17A^+^ with (5). Representative flow cytometry data from one animal is shown. Red numbers indicate percentages of cytokine-producing CD4^+^ T-cell subsets. **(B)** Boxplots summarize the data of experiments with PBMCs from six pigs and show the frequencies of total IFN-γ, total TNF-α, and total IL-17A producing CD4^+^ T cells for the different DON and DOM-1 concentrations. **(C,D)** Boxplots (n = 6) present the frequencies of single IFN-γ, single TNF-α, single IL-17A **(C)**, IFN-γ^+^TNF-α^+^, and TNF-α^+^IL-17A^+^ CD4^+^ T cells **(D)**. Different letters on boxplots indicate significant differences (*p* < 0.05).

To investigate whether increases in transcription factor expression coincided with elevated cytokine production, ConA-stimulated PBMC were treated for 4 h with PMA/ionomycin and CD4^+^ T cells were analyzed for production of IFN-γ, TNF-α, and IL-17A (Figure 2A). In accordance with increased levels of T-bet expression, the frequencies of total IFN-γ and TNF-α producing CD4^+^ T cells were significantly increased at 0.8 μM of DON compared to the other three conditions tested (Figure 2B). Such significant rises were also observed for CD4^+^ T cells that produced only IFN-γ or TNF-α (Figure 2C) or a combination of IFN-γ and TNF-α (Figure 2D). No increases were found for total or single IL-17A-producing CD4^+^ T cells (Figures 2B,C), whereas TNF-α/IL-17A co-producing cells were significantly enhanced for 0.8 μM of DON in comparison to the control (no DON/DOM-1) or 0.2 μM of DON (Figure 2D).

**FIGURE 3:**
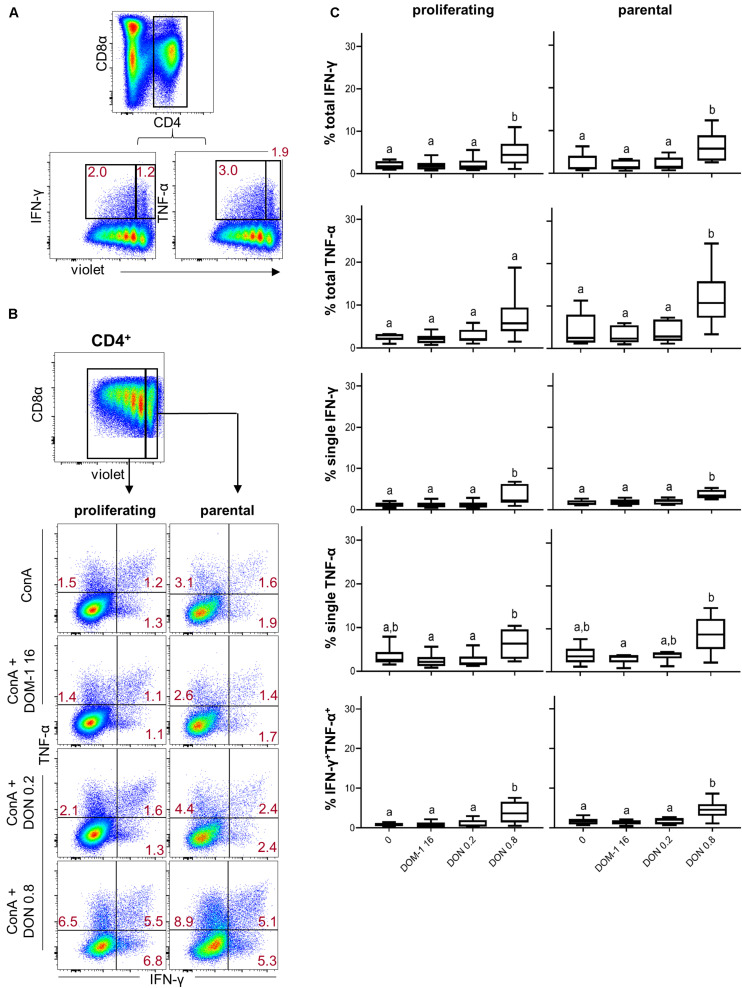
Frequencies of IFN-γ and TNF-α producing cells in proliferating and non-proliferating CD4^+^ T cells in the presence of DON and DOM-1. Violet proliferation dye-stained PBMCs were cultivated for 4 days in the presence of ConA alone or in combination with two different DON concentrations (0.2 and 0.8 μM) and DOM-1 (16 μM). Following PMA/Ionomycin stimulation for 4 h on the fourth day, the cells were harvested and analyzed for cytokine production. **(A)** Representative flow cytometry data showing the gating of CD4^+^ T cells and analysis of total IFN-γ and TNF-α producing cells within proliferating (cells gated on the left) and non-proliferating (cells gated on the right) CD4^+^ T cells. Red numbers give percentages of cytokine producing cells. **(B)** Representative flow cytometry data showing the gating of proliferating and non-proliferating CD4^+^ T cells with subsequent analysis of IFN-γ single, TNF-α single, and IFN-γ/TNF-α co-producing CD4^+^ T cells in the presence of DON and DOM-1. Red numbers indicate percentages of cytokine-producing CD4^+^ T-cell subsets. **(C)** Boxplots summarize the data of experiments with PBMCs from six individual pigs and present the frequencies of proliferating and non-proliferating (parental) CD4^+^ T cells for the different cytokine producing subsets in the presence of DON and DOM-1. Different letters on boxplots indicate significant differences (*p* < 0.05).

We also investigated cytokine production in combination with proliferation. This was done either for all CD4^+^ T cells producing a particular cytokine (“total,” representative data in Figure 3A) or those ones that produced a combination of IFN-γ and TNF-α or just a single cytokine (representative data in Figure 3B). Within proliferating CD4^+^ T cells, a significant increase of total IFN-γ producing cells was found at 0.8 μM of DON in comparison to the other three conditions (Figure 3C, left panel, first diagram from top). The same applied for proliferating single IFN-γ producing and IFN-γ/TNF-α co-producing CD4^+^ T cells (Figure 3C, left panel, second and fifth diagram from top, respectively). For proliferating single TNF-α-producing CD4^+^ T cells a significant difference was found for 0.8 μM of DON in comparison to DOM-1 and 0.2 μM of DON (left panel, fourth diagram from top). Within parental CD4^+^ T cells, 0.8 μM of DON led to a significant rise in cytokine producing cells in comparison to the other three conditions for total IFN-γ and TNF-α, single IFN-γ, and IFN-γ/TNF-α co-producing CD4^+^ T cells (Figure 3C, right panel, first, second, third, and fifth diagram from top, respectively).

**FIGURE 4:**
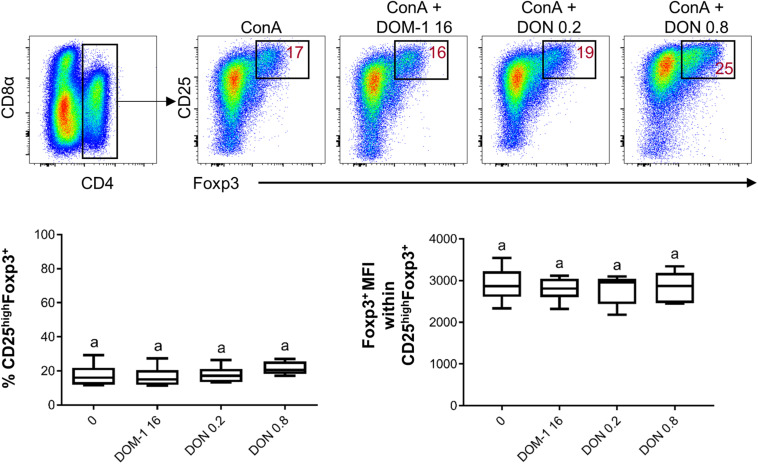
Influence of DON and DOM-1 on the frequency of CD4^+^CD25^high^Foxp3^+^ Tregs and Foxp3 expression. Violet proliferation dye-stained PBMC were cultivated for 4 days in the presence of ConA alone or in combination with two different DON concentrations (0.2 and 0.8 μM) and DOM-1 (16 μM). After harvest, they were labeled for CD4, CD8α, CD25, and Foxp3. Total CD4^+^ T cells were gated and further sub-gated for identification of Tregs (CD25^high^Foxp3^+^). Representative flow cytometry data from one animal is shown. Red numbers indicate percentages of CD25^high^Foxp3^+^ cells. The boxplots show Treg frequencies (CD25^high^Foxp3^+^, left box plot) and Foxp3 expression levels within CD25^high^Foxp3^+^ Tregs (right box plot) in the presence of DON (0.2 and 0.8 μM) and 16 μM of DOM-1 (left). Data was obtained with PBMCs from six individual pigs. No significant differences were observed (*p* > 0.05).

Next, we investigated whether in our *in vitro* system the presence of DON had an influence on the frequency of Tregs (defined by a CD4^+^CD25^high^Foxp3^+^ phenotype) or the expression level of the transcription factor Foxp3 (Figure 4). However, DON treatment did neither increase the frequency of Tregs nor Foxp3 expression.

### Influence of DON on Transcription Factor Expression and Cytokine Production in Porcine CD8^+^ T Cells

**FIGURE 5:**
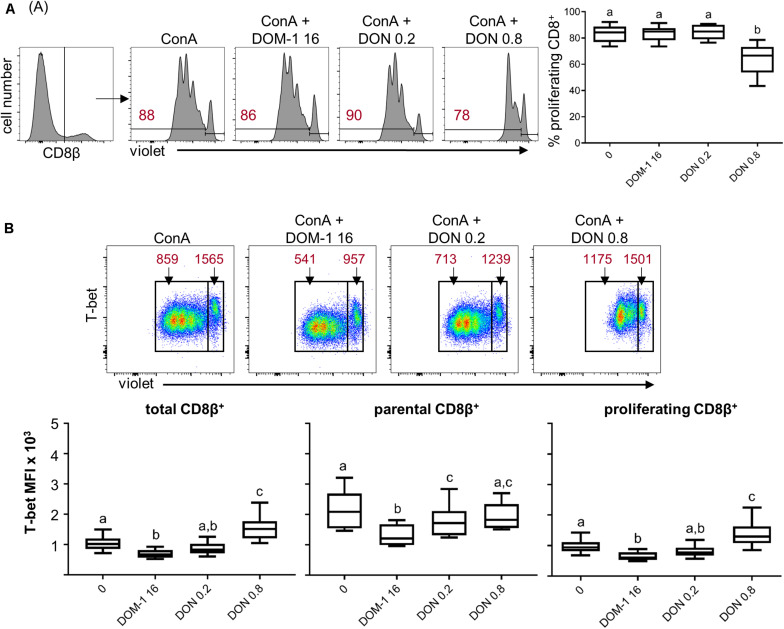
Proliferation and expression of T-bet in CD8^+^ T cells in the presence of DON and DOM-1. Violet proliferation dye-stained PBMC were cultivated for 4 days the presence of ConA alone or in combination with two different DON concentrations (0.2 and 0.8 μM) and DOM-1 (16 μM). After harvest, they were labeled for CD8β and T-bet. **(A)** Flow cytometry panel: representative gating of total CD8β^+^ T cells and raw data of proliferating CD8^+^ T cells under the different conditions. Red numbers show the percentage of proliferating cells. The boxplot on the right displays the percentage of proliferating CD8^+^ T cells for the different DON and DOM-1 conditions. Data was obtained with PBMCs from six individual pigs. **(B)** Analysis of T-bet expression: flow cytometry pseudocolor plots show representative raw data and gates that were applied to identify proliferating (cells gated on the left) and non-proliferating (parental, cells gated on the right) cells. Red numbers indicate median fluorescence intensity (MFI) values for proliferating and non-proliferating cells. Box plots show T-bet expression levels as MFI for total (left) parental (middle) and proliferating (right) CD8^+^ T cells. Data was obtained with PBMCs from six individual pigs. Different letters on boxplots indicate significant differences (*p* < 0.05).

In parallel to CD4^+^ T cells, we investigated the influence of DON on T-bet expression and IFN-γ/TNF-α production in CD8^+^ T cells. Total CD8^+^ T cells were identified by gating on CD8β expressing cells and proliferation was analyzed. As shown previously (5), a DON concentration of 0.8 μM significantly decreased proliferation of CD8^+^ T cells in comparison to cells stimulated in the absence of DON, or in the presence of DOM-1 (16 μM) or 0.2 μM of DON (Figure 5A). The analysis of T-bet expression under these conditions revealed that T-bet was significantly increased by the presence of 0.8 μM of DON in total and in proliferating CD8^+^ T cells compared to the other three conditions. No such increase was found for parental CD8^+^ T cells (Figure 5B). Of note, the obtained results indicate that DOM-1 at a concentration of 16 μM decreased T-bet expression for total, proliferating and parental CD8^+^ T cells in comparison to CD8^+^ T cells cultivated without DON or DOM-1. However, a tendency for such a decrease was also found for 0.2 μM of DON, although significance against no DON or DOM-1 was only reached for parental CD8^+^ T cells.

**FIGURE 6:**
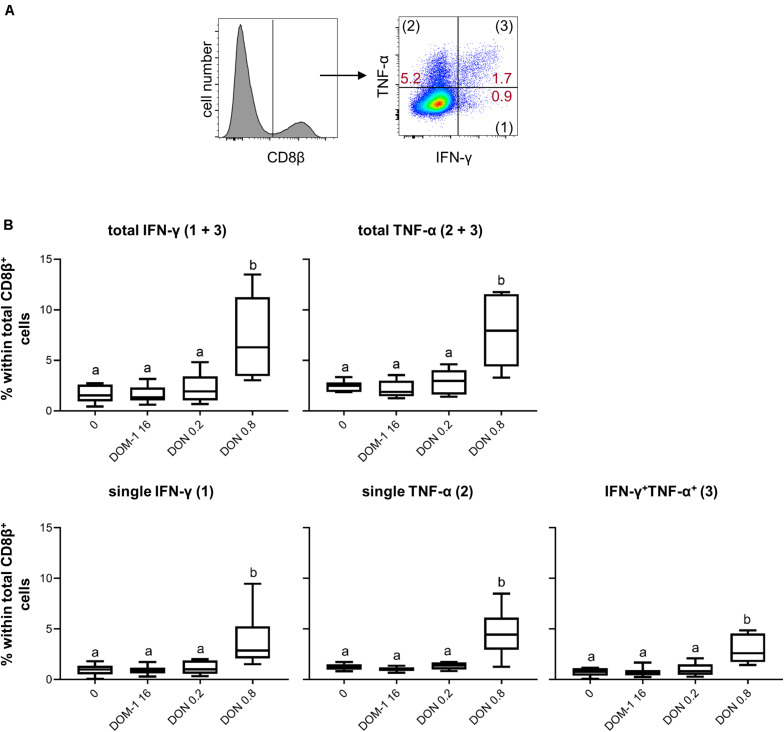
Frequencies of IFN-γ and TNF-α producing CD8^+^ T cells in the presence of DON and DOM-1. Violet proliferation dye-stained PBMCs were cultivated for 4 days in the presence of ConA alone or in combination with two different DON concentrations (0.2 and 0.8 μM) and DOM-1 (16 μM). Following PMA/Ionomycin stimulation for 4 h on the fourth day, the cells were harvested and analyzed for cytokine production. **(A)** Total CD8β^+^ T cells were gated and analyzed for production of IFN-γ and TNF-α. IFN-γ single producing T cells are designated with (1), TNF-α single with (2), and IFN-γ^+^TNF-α^+^ with (3). Representative flow cytometry data from one animal is shown. Red numbers indicate percentages of cytokine-producing CD8^+^ T-cell subsets. **(B)** The boxplots summarize the data of experiments with PBMCs from six individual animals and show the frequencies of total IFN-γ, total TNF-α, single IFN-γ, single TNF-α, and IFN-γ^+^TNF-α^+^ producing CD8^+^ T cells. Different letters on boxplots indicate significant differences (*p* < 0.05).

**FIGURE 7:**
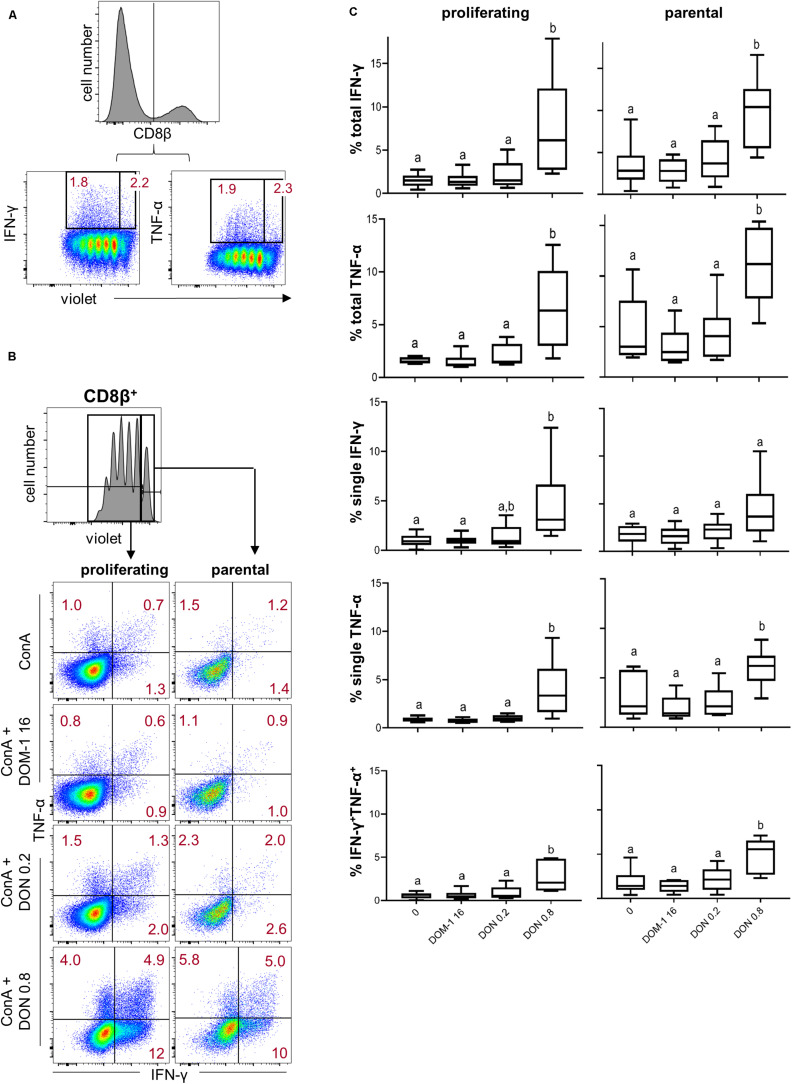
Frequencies of IFN-γ and TNF-α producing cells in proliferating and non-proliferating CD8^+^ T cells in the presence of DON and DOM-1. Violet proliferation dye-stained PBMCs were cultivated for 4 days in the presence of ConA alone or in combination with two different DON concentrations (0.2 and 0.8 μM) and a range of DOM-1 (16 μM). Following PMA/Ionomycin stimulation for 4 h on the fourth day, the cells were harvested and analyzed for cytokine production. **(A)** Representative flow cytometry data showing the gating of CD8β^+^ T cells and analysis of total IFN-γ and TNF-α producing cells within proliferating and non-proliferating CD8 T cells. Red numbers give percentages of cytokine producing cells. **(B)** Representative flow cytometry data showing the gating of proliferating (cells gated on the left) and non-proliferating (cells gated on the right) CD8^+^ T cells with subsequent analysis of IFN-γ single, TNF-α single, and IFN-γ/TNF-α co-producing CD8^+^ T cells in the presence of DON and DOM-1. Red numbers indicate percentages of cytokine-producing CD8^+^ T-cell subsets. **(C)** Boxplots summarize the data of experiments performed with PBMCs of six individual pigs and present the frequencies of proliferating and non-proliferating (parental) CD8^+^ T cells for the different cytokine producing subsets in the presence of DON and DOM-1. Different letters on boxplots indicate significant differences (*p* < 0.05).

Next to T-bet expression, we investigated IFN-γ and TNF-α production in CD8^+^ T cells under the same experimental conditions as for CD4^+^ T cells (Figure 6A). A DON concentration of 0.8 μM resulted in a significant increase in total IFN-γ and TNF-α producing CD8^+^ T cells in comparison to the other three conditions tested (Figure 6B). The same significant rises were found for CD8^+^ T cells that produced only IFN-γ, only TNF-α, or the combination of both cytokines (Figure 6B). We further dissected these findings by investigating also proliferating versus non-proliferating CD8^+^ T cells. This distinction was applied to total IFN-γ and TNF-α producing CD8^+^ T cells (Figure 7A) as well as CD8^+^ T cells that produced only one of the two cytokines or a combination of both (Figure 7B). For all investigated phenotypes of proliferating IFN-γ and TNF-α producing CD8^+^ T cells, we found a significant increase in the frequency of cytokine producing cells for cultures treated with 0.8 μM of DON in comparison to the other three conditions tested (Figure 7C, diagrams on the left panel). Only for proliferating single IFN-γ-producing cells, the difference between 0.2 and 0.8 μM of DON was not significant (Figure 7C, diagrams on the left panel, third diagram from top). For CD8^+^ T cells that did not proliferate, a significant rise in cytokine producing cells in the presence of 0.8 μM of DON was found in comparison to the three other conditions and all cytokine producing subsets investigated (Figure 7C, diagrams on the right panel).

In summary, these results indicate that under the applied stimulation conditions for T cells, 0.8 μM of DON increases T-bet expression in CD4^+^ and CD8^+^ T cells and this increased level of T-bet expression coincides with higher frequencies of cells being capable of the production of IFN-γ and/or TNF-α.

### Influence of DON on Transcription Factor Expression and Cytokine Production in Porcine γδ T Cells

T cells with a γδ TCR are a prominent T-cell subset in porcine blood (32). We could previously show that the proliferation of this T-cell population in the presence of ConA and 0.8 μM of DON is impaired in a similar manner as in CD4^+^ and CD8^+^ T cells (5). In these experiments, expression of the co-stimulatory molecule CD27 was less affected in γδ T cells than in CD4^+^ and CD8^+^ T cells (5), suggesting that γδ T cells may not be influenced by DON in the same way as αβ T cells. Previously, it has been suggested that CD2 expression separates two major subsets of porcine γδ T cells (33). In addition, it was shown that *ex vivo* CD2^–^ γδ T cells express high levels of GATA-3, whereas CD2^+^ γδ T cells partially express T-bet (34). Hence, for the present study, we investigated the influence of DON and DOM-1 on the expression of the transcription factors GATA-3 and T-bet in γδ T cells in combination with CD2.

**FIGURE 8:**
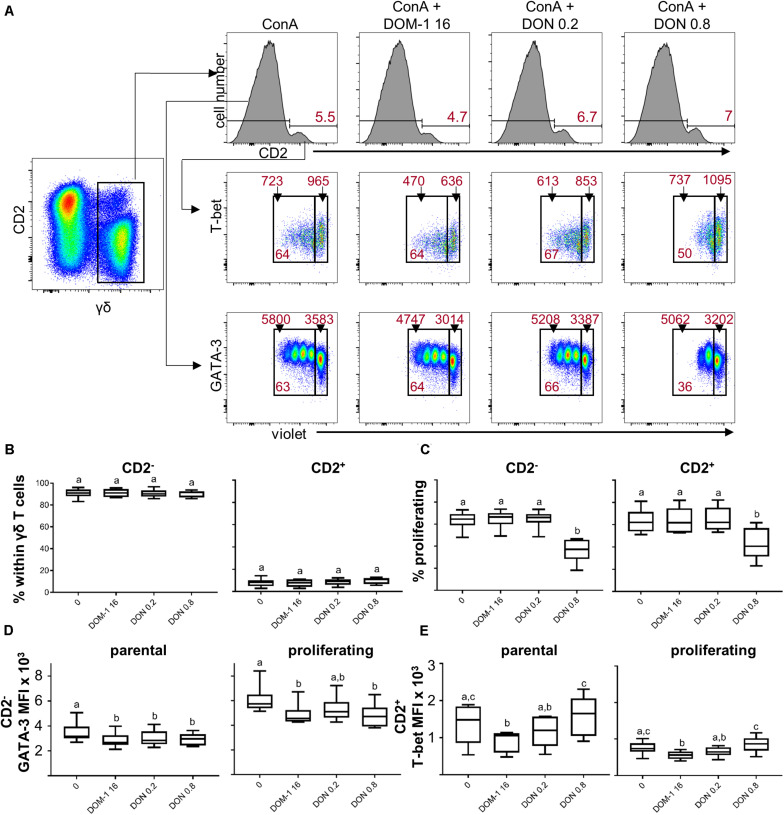
Influence of DON and DOM-1 on proliferation, CD2, GATA-3 and T-bet expression in γδ T cells. Violet proliferation dye-stained PBMC were cultivated for 4 days in the presence of ConA alone or in combination with two different DON concentrations (0.2 and 0.8 μM) and DOM-1 (16 μM). After harvest, they were stained for TCR-γδ, CD2, T-bet, and GATA-3. **(A)** γδ T cells were gated for identification of CD2^+^ and CD2^–^ γδ T cells (histograms, top row). Within CD2^+^ γδ T cells T-bet was analyzed in combination with proliferation and proliferating (cells gated on the left) and non-proliferating (cells gated on the right) subsets were gated (middle panel). The same was applied to CD2^–^ γδ T cells in combination with GATA-3 expression (bottom panel). Representative flow cytometry data from one animal is shown. In the top panel, red numbers give percentages of CD2^+^ γδ T cells. In the middle and bottom panel, red numbers on top of gates indicate median fluorescence intensity (MFI) values, whereas red numbers in the gates indicate percentages of proliferating cells. **(B–E)** Boxplots show the results for γδ T cells within PBMCs from six individual pigs. **(B)** Frequencies of CD2^–^ and CD2^+^ cells within γδ T cells. **(C)** Proliferation of CD2^–^ and CD2^+^ cells within γδ T cells. **(D)** Expression of GATA-3 within CD2^–^ γδ T cells separated into parental and proliferating subsets **(E)** Expression of T-bet within CD2^+^ γδ T cells separated into parental and proliferating subsets. Different letters on boxplots indicate significant differences (*p* < 0.05).

We initially investigated whether DON or DOM-1 influence the relative proportion of ConA-stimulated CD2^+^ and CD2^–^ γδ T cells. Hence, total γδ T cells were gated and analyzed for CD2 expression (Figure 8A, top row). CD2^–^ γδ T cells clearly dominated over CD2^+^ γδ T cells, but their relative proportion to each other was not influenced by the investigated DON or DOM-1 concentrations (Figure 8B). When these two subsets were analyzed for proliferation, a significant reduction in proliferating cells was found in the presence of 0.8 μM of DON, whereas DOM-1 (16 μM) or 0.2 μM of DON did not affect ConA-induced proliferation (Figure 8C). Analysis of GATA-3 expression in proliferating and non-proliferating (parental) CD2^–^ γδ T cells (see Figure 8A, bottom panel, for representative raw data) revealed that in parental CD2^–^ γδ T cells both DON concentrations and DOM-1 caused a significant reduction in GATA-3 expression levels (Figure 8D). Similarly, in proliferating CD2^–^ γδ T cells a significant reduction of GATA-3 was found for 0.8 μM DON and 16 μM DOM-1. GATA-3 was also diminished at 0.2 μM DON, but significance was not reached. For T-bet expression within CD2^+^ γδ T cells (representative raw data shown in the middle panel of Figure 8A), a significant reduction in T-bet expression was found with 16 μM of DOM-1 in comparison to the ConA-only condition in both parental and proliferating γδ T cells (Figure 8E). T-bet expression in CD2^+^ γδ T cells at a DON concentration of 0.2 μM was significantly lower than in DON 0.8 μM treated cultures. However, different from CD4^+^ and CD8^+^ T cells, there was no significant increase of T-bet expression at 0.8 μM of DON in comparison to the ConA-only condition. This applied both to parental and proliferating γδ T cells.

**FIGURE 9:**
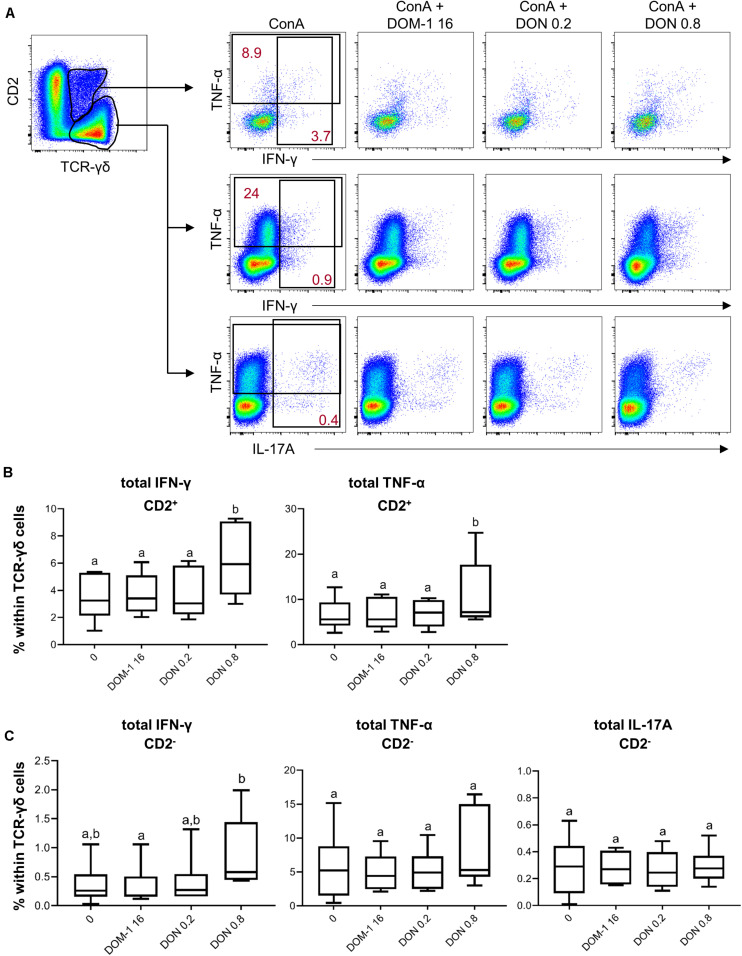
Frequencies of IFN-γ, TNF-α, and IL-17A producing CD2^+^ and CD2^–^ γδ T cells in the presence of DON and DOM-1. Violet proliferation dye-stained PBMCs were cultivated for 4 days in the presence of ConA alone or in combination with two different DON concentrations (0.2 and 0.8 μM) and DOM-1 (16 μM). Following PMA/Ionomycin stimulation for 4 h on the fourth day, the cells were harvested and analyzed for cytokine production. **(A)** CD2^+^ and CD2^–^ γδ T cells were gated and further analyzed for production of TNF-α, IFN-γ, and IL-17A in the presence of DON and DOM-1. The black rectangles in the pseudocolor plots show the gating strategy that was applied for analysis of total IFN-γ, total TNF-α, and total IL-17A producing cells. Red numbers give percentages of cytokine producing γδ T cells in these gates. Representative flow cytometry data from one animal is shown. **(B,C)** Boxplots show the results for γδ T cells within PBMCs from six individual pigs. **(B)** Frequency of IFN-γ and TNF-α producing cells within CD2^+^ γδ T cells. **(C)** Frequency of IFN-γ, TNF-α, and IL-17A producing cells within CD2^–^ γδ T cells. Different letters on boxplots indicate significant differences (*p* < 0.05).

Following stimulation with PMA/ionomycin, porcine γδ T cells are capable of producing IFN-γ, TNF-α, and IL-17A, with IFN-γ production strongly dominating in CD2^+^ γδ T cells, whereas a small subset of CD2^–^ γδ T cells has the capacity to produce IL-17A (35). Hence, we analyzed the production of these three cytokines in CD2^+^ and CD2^–^ γδ T cells (Figure 9A shows gating strategy and representative raw data). Since the frequencies of IFN-γ and IL-17A producing γδ T cells were rather low, we decided to analyze only total cytokine producing cells for one particular cytokine, but did not investigate single- or cytokine co-producing cell subsets. For CD2^+^ γδ T cells a significant rise in IFN-γ and TNF-α producing cells was found in the presence of 0.8 μM of DON in comparison to the other three conditions (Figure 9B). For CD2^–^ γδ T cells, only IFN-γ producing cells differed significantly between 16 μM of DOM-1 and 0.8 μM of DON (Figure 9C).

## Discussion

In the present study, we analyzed the influence of DON on the expression of transcription factors and related cytokines within porcine T cells by making use of *in vitro* stimulated PBMC cultures. Over the last years, it has become clear that by binding to the ribosome DON influences the cell metabolism in many different ways (11, 12, 14). After appropriate stimulation, cells of the adaptive immunes system first undergo several rounds of proliferation and then differentiate in effector cells, which usually have a high metabolic activity (36). Hence, we assumed that *in vitro* cultures of porcine PBMC, which we stimulated with the polyclonal agent ConA are a useful model to study the influence of DON on activated T cells. In this way, we expected to elucidate mechanisms on how DON affects T-cell transcription factor expression and the production of related cytokines, which are both indicative of the function that such cells may perform *in vivo*.

For CD4^+^ and γδ T cells, we investigated the expression of T-bet and GATA-3, along with the cytokines IFN-γ, TNF-α, and IL-17A. This selection was made based on previous studies which showed that these two porcine T-cell subsets express these transcription factors (34, 37) and contain subpopulations that produce these cytokines after polyclonal stimulation (26, 35). In contrast, production of IL-4 and IL-10 is usually low in PMA/ionomycin or ConA-stimulated cultures of porcine PBMC (38). For porcine CD8^+^ T cells, we investigated T-bet and the cytokines IFN-γ and TNF-α, but excluded GATA-3 and IL-17A. So far GATA-3 expression could not be identified in extrathymic CD8^+^ T cells of pigs (37). In addition, no IL-17A production was found so far in this T-cell population (our unpublished findings).

For the presented experiments, we selected a concentration of 0.8 μM DON. This concentration was chosen because in previous studies with the same experimental set-up (5) it was found that 0.8 μM DON reduces proliferation of porcine CD4^+^, CD8^+^, and γδ T cells but does not entirely abolish it. Hence, we hypothesized that at this concentration DON may exert also other effects. Next to 0.8 μM DON, we tested a lower DON concentration of 0.2 μM. In our previous study, this concentration did not affect proliferation or the expression of co-stimulatory molecules (5), but we speculated that such a concentration might affect other functional traits of T cells. Moreover, feed contaminated with as little as 200 μg/kg DON, which is equivalent to approximately 0.6 μM, translates already into detectable levels of DON in the blood (39), illustrating the practical relevance of the two tested DON concentrations. For the microbial transformation product of DON, designated as DOM-1, a 20-fold higher concentration than for DON was investigated. This substantially higher concentration was selected because in our previous study DOM-1 did not affect proliferation or the expression of the T-cell costimulatory molecules CD27 and CD28 (5). Moreover, previous work indicated that DOM-1 has lost its capacity to induce the activation of MAPKs in human intestinal cells and porcine jejunal cell explants (14).

For CD4^+^ and CD8^+^ T cells, we found increased expression levels of T-bet in particular in proliferating cells in the presence of 0.8 μM of DON. This coincided with increased frequencies of IFN-γ and TNF-α but not IL-17A producing cells. T-bet expression is induced by the polarizing cytokines IL-12 and IFN-γ (20). However, this requires in-between the action of the transcription factors NFAT, AP-1, and STAT-1, which drive T-bet expression (40). As mentioned above, when bulk cultures of porcine PBMC are stimulated with polyclonal agents like PMA/ionomycin or ConA, the production of IFN-γ is often much higher in comparison to other cytokines like IL-4, IL-17A, or IL-10 (26, 34, 38). Hence, it is likely that the ConA stimulation led to increased levels of IFN-γ in the PBMC microcultures from early on after the start of cultivation. For DON it has been described that – among other MAPKs – it rapidly induces Erk1/2 and p38, both in macrophage cell lines (41) but also in the Jurkat cells (42), which are of T-cell origin. These MAPKs are also activated following TCR triggering and can drive in combination with calcium flux the expression of NFAT and AP-1 (43). Hence, it is conceivable that a ConA-driven TCR-stimulation is further accelerated by DON, leading to high levels of NFAT and AP-1, which together with IFN-γ-induced STAT-1 drive T-bet expression. This T-bet expression would then further enhance IFN-γ production because of the dominance of a Th1-priming milieu. This may have applied in particular to naïve T cells, which have a high capacity for proliferation (44) and can still be driven by cytokines in particular directions of functional differentiation (23). In addition, in metabolically active cells such as those ones undergoing proliferation, ribosomal activity is also high, which may further increase the impact of DON. The postulated accelerator mechanism of DON on T-cell differentiation in the context of a Th1 polarizing cytokine milieu would also explain why there was no increase in the frequency of IL-17A producing CD4^+^ (and potentially γδ) T cells as well as Foxp3-expressing Tregs. To test this hypothesis, future experiments could make use of sorted naïve CD4 T cells and then test the influence of DON on T-cell differentiation with combinations of polarizing cytokines, identified in experiments with murine and human T cells (23).

However, somewhat in contradiction to the arguments raised above, not only for T-bet but also for GATA-3 an up-regulation was observed in proliferating CD4^+^ T cells. This might be explained by the observation that at least during thymic development GATA-3 expression raises during stages of high proliferation (45). Moreover, it has been reported that a polyclonal stimulation of CD4^+^ T cells increases GATA-3 expression (46). Hence, under the influence of ConA DON may again increase further the expression of a transcription factor, which is already induced by the polyclonal stimulation of the T cells in the PBMC-cultures.

Whereas CD4^+^ and CD8^+^ T cells gave similar results in our experiments (upregulation of T-bet in the presence of 0.8 μM DON, increased numbers of IFN-γ and TNF-α producing cells), results in γδ T cells were more variable. In CD2^–^ γδ T cells, GATA-3 expression was reduced in the presence of 0.8 μM DON, but this applied also to the other two conditions tested (16 μM DOM-1, 0.2 μM DON, Figure 8D). In CD2^+^ γδ T cells, T-bet expression levels at 0.8 μM DON were slightly increased but did not differ significantly from the control condition (no DON, no DOM-1, Figure 8E). This coincided with increased IFN-γ and TNF-α production at 0.8 μM of DON in this γδ T-cell subset. However, in CD2^–^ γδ T cells frequencies of IFN-γ, TNF-α, or IL-17A producing γδ T cells were not changed by 0.8 μM DON, at least in comparison to the control cultures. The reasons for these observations remain speculative. Porcine γδ T cells proliferate less vigorously to ConA than CD4^+^ or CD8^+^ T cells (5). Therefore, the influence of DON may be not as prominent due to a lower rate of protein translation at the ribosome. However, at least for CD4^+^ T cells there was an upregulation of T-bet even in non-proliferating cells (Figure 1B). Another reason might be the hitherto uncharacterized cytokine requirements that drive the differentiation of porcine γδ T cells. We could recently show that a cocktail of IL-2, IL-12, and IL-18 induces T-bet expression in proliferating CD2^–^ γδ T cells, while these cells keep a high expression of GATA-3 (34). However, IL-12 is hardly induced in porcine PBMC by polyclonal stimulation (38). Hence, it is probably not justified to conclude that γδ T cells are less affected by DON. Instead, our experimental conditions may not have revealed such effects.

In some of our experiments the high concentration of DOM-1 (16 μM) caused a loss in T-bet expression (Figures 5B, 8E). Despite its remaining capacity to bind to the ribosome, DOM-1 has been described to have a substantially reduced toxicity and activity, as seen for oxygen consumption, barrier function and MAPK induction in human intestinal epithelial cells at a concentration of 10 μM in comparison to DON (14). In addition, our previous experiments with 16 μMDOM-1 did not reveal a loss of proliferation or the expression of co-stimulatory molecules in porcine T cells (5). However, more recently it was shown by *in vivo* experiments that DOM-1 can support cell proliferation in lymph nodes and antibody production to the model antigen ovalbumin to a similar extent as DON (47). Also, a study with bovine theca cells indicated that DOM-1 can increase mRNA expression of endoplasmatic reticulum stress-related proteins (48). This suggests that DOM-1 has still some biological activity, at least at such high concentrations as used in our study. Next to these findings, Novak et al. (4) mentioned that in the DOM-1 that was used in their *in vitro* experiments 0.1–0.2% impurities of DON were detected that could explain some of the immunomodulatory effects of the tested substance.

Our study elucidates some of the underlying mechanisms how DON might influence functional properties of T cells *in vivo*. Related to these *in vitro* observations presented here, recent *in vivo* experiments from our team indicate that a low concentration of DON in pig feed (0.9 ppm) can increase the frequency of virus-specific IFN-γ/TNF-α co-producing CD4^+^ T cells after a combination of vaccination and challenge infection with porcine reproductive and respiratory syndrome virus (Pierron et al., in preparation). This provides further hints that at non-toxic doses, the capacity of DON to bind to the ribosome may accelerate the translation of proteins that is already ongoing, at least in T cells. Depending on the external stimuli that drive T-cell differentiation, this may have beneficial or detrimental effects on the outcome of the immune response.

## Data Availability Statement

The raw data supporting the conclusions of this article will be made available by the authors, without undue reservation.

## Ethics Statement

Ethical review and approval was not required for the animal study because blood samples were collected from pigs which had been slaughtered at a commercial slaughterhouse for meat consumption. The animals were anesthetized with high electric voltage, which was followed by exsanguination, a procedure which is in accordance to the Austrian Animal Welfare Slaughter Regulation.

## Author Contributions

WG, AS, and EM designed the study and acquired the funding. EV performed the proliferation assays and transcription factor analyses. AP and AH performed the experiments for intracellular cytokine staining. EV, AP, AH, and WG analyzed the data. EV and WG wrote the manuscript. All authors edited the manuscript and agreed on the final version.

## Conflict of Interest

EM is employed by BIOMIN, which operates the BIOMIN Holding GmbH, which is a producer of animal feed additives. This, however, did not influence the design of the experimental studies or bias the presentation and interpretation of results. The remaining authors declare that the research was conducted in the absence of any commercial or financial relationships that could be construed as a potential conflict of interest.
