# Supplementary material for: Deoxynivalenol Has the Capacity to Increase Transcription Factor Expression and Cytokine Production in Porcine T Cells
Source: Front Immunol. 2020 Aug 13;11:2009. doi: 10.3389/fimmu.2020.02009 (PMC7438481; doi:10.3389/fimmu.2020.02009)
Supplement: Supplementary file 1 [file Data_Sheet_1.docx]

Supplementary Material

**Deoxynivalenol has the capacity to increase transcription factor expression and cytokine production in porcine T cells**

**Eleni Vatzia, Alix Pierron, Anna Maria Hoog, Armin Saalmüller, Elisabeth Mayer, Wilhelm Gerner**

# Supplementary Figures

**Supplementary Figure 1. Representative gating strategy of lymphocytes cultivated in the presence of ConA.** PBMCs were cultivated for four days in the presence of ConA alone or in combination with different DON and DOM-1 concentrations. After harvest, lymphocytes (including blast cells) were gated according to their light scatter properties, followed by gating on live cells. In the shown example, CD4^+^ T cells were gated within live lymphocytes. The same gating hierarchy was applied to PBMC that had undergone PMA/ionomycin stimulation 4 hours prior to harvest.
